# Artificial Intelligence and Finance: A bibliometric review on the Trends, Influences, and Research Directions

**DOI:** 10.12688/f1000research.160959.1

**Published:** 2025-01-23

**Authors:** Prasenjit Roy, Biswajit Ghose, Premendra Kumar Singh, Pankaj Kumar Tyagi, Asokan Vasudevan

**Affiliations:** 1 Department of Commerce, Tezpur University, Napaam, Assam, India; 2 Centre for Distance and Online Education, Sharda University, Greater Noida, Uttar Pradesh, India; 3 Chandigarh University, Mohali, Punjab, India; 4 Faculty of Business and Communications, INTI International University, Nilai, Negeri Sembilan, Malaysia

**Keywords:** Artificial Intelligence, Finance, Bibliometric Analysis, TCCM, Thematic Mapping

## Abstract

**Background:**

This bibliometric study examines the intersection of artificial intelligence (AI) and finance, providing a comprehensive analysis of its evolution, central themes, and avenues for further exploration. The study aims to uncover the theoretical foundations, methodological approaches, and practical implications of AI in financial contexts.

**Methods:**

The research employs bibliometric techniques, using 607 Web of Science (WoS) indexed papers. The Theory-Context-Characteristics-Methodology (TCCM) framework guides the analysis, focusing on thematic mapping to explore key topics. Core areas such as risk management, market efficiency, and innovation are analyzed, alongside emerging themes like ethical AI, finance applications, and factors influencing AI-driven financial decision-making.

**Results:**

The findings reveal critical gaps in interdisciplinary methods, ethical considerations, and methodological advancements necessary to develop robust and transparent AI systems. Thematic mapping highlights the increasing importance of ethical AI practices and the influence of AI on financial decision-making processes. Emerging research areas emphasize the need for innovative frameworks and solutions to address current challenges.

**Conclusions:**

This study provides valuable insights for academics, industry practitioners, and policymakers to harness transformative potential of AI in finance. This research offers a foundation for future studies and practical applications by addressing key gaps and promoting interdisciplinary and ethical approaches in a rapidly evolving field.

## 1. Introduction

Artificial intelligence (AI) has caused significant changes in several industries, among which the finance industry has been the most impacted. AI is defined as the imitation of human intelligence processes by machines, particularly computer systems ( Russell & Norvig, 2016). AI improves decision-making, increases efficiency, and reduces human errors. The convergence of AI and finance, sometimes referred to as Fintech (financial technology), has produced novel tools and models, including automated customer service, credit scoring, and algorithmic trading ( Arner et al., 2016). These advancements, powered by machine learning, deep learning, and natural language processing, have altered customer experiences and market dynamics, in addition to improving the accuracy and speed of financial decision-making ( Chong et al., 2017). Identifying the development and major trends in AI has become crucial for researchers, practitioners, and policymakers alike, as the field’s significance in the financial sector has only increased.

Academic focus on AI in finance has risen over the last 20 years, and as technology develops, this trend is anticipated to continue. Scholars from fields such as computer science, economics, management, and business have made immense contributions. Currently, AI is employed in many financial applications such as automated wealth management, fraud detection, and market behavior prediction ( Brynjolfsson & McAfee, 2014). Machine learning algorithms have proven remarkably effective in predicting stock market trends and identifying patterns in large datasets that cannot be detected by humans ( Henrique et al., 2019; Nabipour et al., 2020; Patil et al., 2024). Similarly, financial institutions may now offer personalized customer support through chatbots and virtual assistants through natural language processing, which improves service quality and lowers costs ( Bock et al., 2020; Engles & Bolze, 2020).

Despite these developments, there are still few systematic studies on the development and research trends in the literature on AI in finance. Existing studies have often focused on specific AI technologies or their applications in particular areas of finance, such as trading or risk management ( Cao, 2023; Dixon et al., 2020). However, there is still a lack of a thorough overview that maps its evolution, highlights its major topics, and describes new paths of inquiry. This gap accentuates the need to highlight AI and finance research using a bibliometric analysis. Applying statistical techniques to the study of scientific literature or bibliometrics can yield important information on the dynamics and organization of the subject of study ( Aria & Cuccurullo, 2017). Bibliometric studies assist in determining the development of a field of study, as well as the relationships between various subjects and subfields, by examining trends in publication volume, citation patterns, and the most significant articles and authors.

The financial industry’s increasing reliance on AI technologies has resulted in both benefits and difficulties. On the one side, AI can increase customer satisfaction, reduce operating expenses, and enhance decision-making. However, there are additional hazards associated with the growing use of AI, including algorithmic biases, concerns about data privacy, and the possibility of job displacement in the financial services sector ( Pal et al., 2021). As AI has become more integrated into the financial industry, research must address these concerns and find strategies to mitigate its negative consequences. Future studies should concentrate on the ethical implications of AI in finance, such as equity in algorithmic decision-making and transparency in the AI model ( Kurshan et al., 2021).

The focus of this article is to fill this vacuum by offering a thorough overview of AI and finance research. The main aim was to determine the most referenced papers, significant authors, and important research issues in this field. The study also seeks to map the evolution of the sector and identify emerging patterns that could affect future use of AI in finance . This research is important because it will provide better knowledge of the academic progress in this field and identify areas for further study. The following research issues were addressed in the study to address the following research questions:

Q1: How has the literature on AI and finance evolved till 2024?

Q2: Which are the most productive journals, important articles, and renowned authors in AI and finance literature?

Q3: What are the main tools, uses, and areas of study that influence the interaction between AI and finance?

Q4: What are the challenges and future paths of AI and finance research?

The findings from this study could aid researchers in gaining a better view of the role of AI in finance, inform practitioners on the latest advancements, and assist policymakers in addressing ethical, operational, and regulatory concerns related to the field. This article is divided into six sections. We outline the process for locating, screening, and choosing relevant publications for the bibliometric study of AI in finance in the first section, which also presents the data and methodology. Using Bibiliometrix and VoSviewer software, we present a bibliometric overview in the second section. We created co-citation networks, cited significant articles and authors, highlighted the most productive journals, and identified publication patterns ( Aria & Cuccurullo, 2017). The final section provides a comprehensive framework for the dominance of AI in the financial sector using the Textual, Contextual, Conceptual, and Methodological (TCCM)paradigm. In addition to offering insights into topics and subdomains, it also helps map the evolution of AI applications in finance. In the fourth segment, we examine the theme and future directions, looking at existing trends, gaps, and potential future research topics. The fifth section offers a synthesis of areas that require more research and opportunities for development as well as a synopsis of future study directions. The sixth section concludes with a review of the research’s limitations and a list of the key lessons learned, and finally discusses the implications of our findings.

## 2. Methods

### 2.1 Data and methodology

We employed a mixed review technique in this study, combining the TCCM framework with bibliometric review to identify research trends and propose future possibilities within the research context. The TCCM framework examines existing theoretical and research frameworks and suggests changes or the creation of new models ( Paul & Rosado-Serrano, 2019). This approach is consistent with the design and content of the accepted review procedures ( Bhukya & Paul, 2023). Furthermore, we incorporated inclusion and exclusion criteria ( Bhukya & Paul, 2023) when choosing the materials for analysis. Articles from peer-reviewed journals in the Web of Science (WoS) database with an impact factor of at least one were included.

The selection follows a three-step process: i) identification of relevant documents, ii) screening of documents, and iii) final selection. This establishes the relevance and rigour of the materials required for the study. The theories, constructions, contexts, and methodologies in the literature were evaluated using the TCCM framework. Bibliometric analysis reveals information on cocitation networks, publishing trends, and citation patterns. We applied an integrated approach, that help us to uncover new topics and knowledge gaps for future research. This provides a broad picture of the evolution of AI applications in finance. This would establish a foundation for future theoretical and methodological advancements and provide useful recommendations for academics and specialists in the area.

### 2.2 Identification

The WoS bibliometric database ( http://www.webofknowledge.com) was used to identify research articles relevant to AI and finance. The search terms included “artificial intelligence” “AI” and “finance” finance, which were applied to conduct title, abstract, and keyword searches. The initial query retrieved a total of n = 2476 documents connected to AI and finance research. Recognizing the possibility that not all documents would be directly relevant to the research scope, further screening was performed using predefined filters to ensure the selection of pertinent documents.

### 2.3 Screening

**Figure 1.**
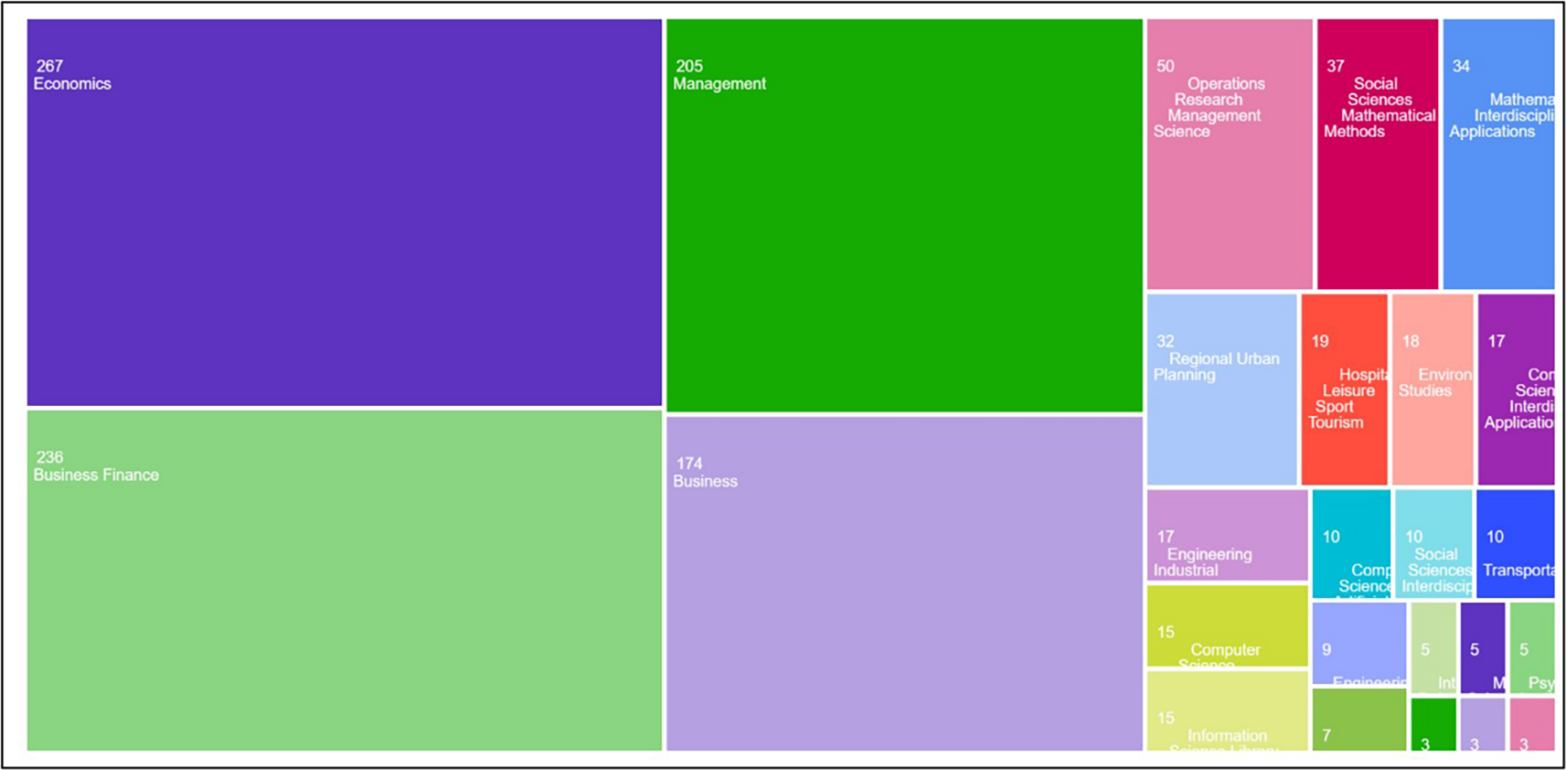
Tree Map indicating initial query results.

A multilayered filtering process was employed to refine the document set and maintain its relevance to the study objectives. First, the scope was narrowed to publications categorized under the fields of ‘Economics,’ ‘Business Finance,’ ‘Management,’ and ‘Business, econometrics, and finance’ as depicted Figure 1. Second, only the final versions of the peer-reviewed journal papers published in English were included. The filtering process returned a reduced dataset of 629 documents. To ensure data quality, additional manual screening was performed to confirm the relevance of these articles to AI and finance research.

### 2.4 Final Inclusion

Following comprehensive screening, 607 documents were finalized as highly relevant to the literature on AI in finance. Manual verification of the dataset was performed and each document provided substantial insights. These documents were included in the subsequent bibliometric analyses.

## 3. Bibliometric overview

Advanced bibliometric analytic tools, such as Bibliometrix ( https://www.bibliometrix.org/home/) and VOSviewer ( https://www.vosviewer.com/), which are frequently used in bibliometric investigations, were employed in this study ( Ingale & Paluri, 2020). A bibliometric analysis was conducted on the finalized dataset to uncover publication trends, influential works, and key contributors to the domain of AI in finance.

a) Trend Analysis: The analysis begins with a trend overview of AI and finance research, highlighting publication growth over time and identifying periods of significant research activity.
b) Most Cited Articles: An overview of the most referenced papers in finance and AI literature is provided, highlighting significant research that has influenced the field.
c) Leading Journals: The study identifies journals with the highest impact, which have published groundbreaking works in AI and its applications in finance.
d) Productive Authors: Finally, the analysis highlights the most prolific and impactful authors who have significantly contributed to AI and finance research.

This methodological approach facilitates insights into existing trends and identifies areas for future research, thus offering extensive knowledge of the research landscape in AI and finance.

### 3.1 Publication trend

Since the mid-1900s, the concept of AI has experienced sizable growth. At the Dartmouth Conference in 1956, John McCarthy first used the word to describe the making of machinery that might carry out assignments that normally required human intelligence ( McCarthy et al., 1956). Over time, AI has been expanded to encompass machine learning, deep learning, and neural networks, moving away from its initial focus on rule-based systems and symbolic reasoning ( Mohamed et al., 2020; Russell & Norvig, 2016). AI’s increasing capacity to evaluate enormous datasets, adjust to new knowledge, and produce remarkably accurate forecast insights is shown in this progression.

AI’s transformative impact has been particularly profound in the financial sector, where its adoption has redefined traditional processes and decision-making paradigms. Financial institutions have leveraged AI for applications ranging from algorithmic trading and fraud detection to personalized customer services and risk assessment ( Agrawal et al., 2018). For instance, AI powered algorithmic trading systems can process and analyze financial market data in real time, enabling high-frequency trades that maximize profitability ( Baldauf & Mollner, 2020). Similarly, financial institutions can anticipate market patterns, identify possible weaknesses, and create plans to reduce losses by using AI-driven risk management systems ( Cao, 2023; Jain, 2023).

AI is essential for improving customer engagement and personalizing experiences in marketing and consumer finance. AI-powered advanced recommendation engines examine consumer behavior and preferences in order to make personalized financial product recommendations, enhance client loyalty, and foster stronger relationships ( Davenport & Ronanki, 2018). Furthermore, chatbots and virtual assistants provide real-time assistance with natural language processing (NLP) capabilities to revolutionize customer service in banking and financial services ( Coglianese & Lehr, 2019; Shin, 2019). Recent bibliometric analyses of AI in finance literature stresses its interdisciplinary nature and rapid expansion as a field of study. For instance, studies have identified key trends in AI applications within financial services, highlighting an emphasis on predictive analytics, real-time decision making, and automation ( Dhamija & Bag, 2020). Bibliometric research has also revealed the central role of collaboration among researchers from diverse disciplines, thus reflecting the field’s complex and integrative challenges. In this study we have further explored the ethical considerations and governance challenges accompanying AI’s widespread adoption in finance, emphasizing the importance of balancing innovation with regulatory oversight ( Li & Zhang, 2024; Ridzuan et al., 2024; Taeihagh, 2021).

AI is a key force behind innovation in the financial industry because of its capacity to interpret unstructured data, identify hidden patterns, and make judgments on its own. Meanwhile, debates over fairness, transparency, and the possible replacement of human labor in finance-related positions have been sparked by its broad implementation ( Cao, 2023; Pal et al., 2021). As AI develops further, its dual function as a tool to improve operational efficiency and ethical investigation highlights its great and complex effect on finance.

**Figure 2.**
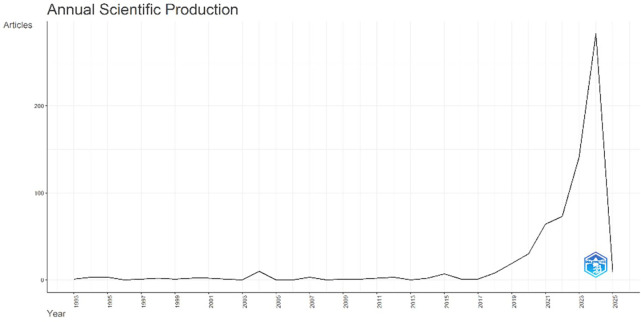
Annual scientific production.

The status of scientific production for each year is shown in Figure 2 and Table 1. Our bibliometric research documents the scholarly investigation of AI in finance, identifying 607 papers published between 1993 and 2024, starting in the early 1990s. This area has shown consistent improvement, with a yearly growth rate of 3.49%. Throughout the first 25 years (1993–2017), academic output was relatively modest, with only 139 publications, averaging approximately 5.56 articles annually. However, this changed dramatically after 2018, with an exponential increase in the publication rates. From 2018 to 2023 alone, an average of 121.6 publications per year demonstrates the accelerating interest in AI applications within finance. The majority of research contributions result from this increase, suggesting that in recent years, the relationship between AI and finance has become a crucial topic of study.

**Table 1.** Description of publication trend.

Description	Results
MAIN INFORMATION ABOUT DATA	
Timespan	1993:2025
Sources (Journals, Books, etc.)	205
Documents	607
Annual Growth Rate %	3.49
Document Average Age	2.98
Average citations per doc	15.73
References	30726
DOCUMENT CONTENTS	
Keywords Plus (ID)	1407
Author's Keywords (DE)	2303
AUTHORS	
Authors	1577
Authors of single-authored docs	45
AUTHORS COLLABORATION	
Single-authored docs	46
Co-Authors per Doc	3.6
International co-authorships %	45.63

Source: Compiled by Authors using VoSviewer.

An average of 15.73 citations per document and 30,726 references showed a growing volume of research in the area. Furthermore, international collaboration is prominent in this domain, with 45.63% of publications involving authors from multiple countries highlighting the global relevance of AI in reshaping financial systems. Major applications explored in the literature include risk management, trading algorithms, customer relationship management, and decision-making tools. Scholarly interest increased sharply between 2019 and 2023, coinciding with advances in automation, big data analytics, and machine learning. The relatively young age of documents (2.98 years on average) suggests that this is an emergent field, characterized by fresh insights and cutting-edge research. Despite the recent proliferation of studies, the field is still in its formative stages, with single-authored contributions comprising only 46 articles, indicating the collaborative nature of this research domain. The expanding adoption of AI technologies by financial institutions and fintech companies, along with the emergence of data-driven solutions, is expected to sustain and even amplify this growth trajectory. The sharp increase in publications after 2018 highlights the transformative capability in this area. As the financial sector continues to embrace AI-driven innovations, further academic exploration is anticipated, promising to enhance our insight into the role of AI in revolutionizing financial systems and practices.

### 3.2 Productive Journals

The breakdown showed that 607 articles were published in 205 journals. We searched for journals with at least one publication and at least 25 citations to determine which were the most significant and active in furthering AI and finance research. 64 journals that satisfied the required limits were identified using this criterion ( Table 2). The journals were ranked according to the quantity of publications they published; the journal that published the most articles was ranked the highest. The rating was based on the overall number of citations earned by each publication, even if several journals had published the same number of articles. Our analysis indicates that *Management Science, Journal of Banking & Finance, Technological Forecasting and Social Change, Finance analysis Letters, and Journal of Economic Dynamics & Control* are the top five most productive publications for AI and finance research. The advancement of the literature in this field has been greatly aided by these journals. According to the ABDC journal rankings, these journals are notably ranked A or A*, demonstrating their significance and the significant influence of the research they publish. Table 2 provides a thorough summary of the journals, along with their citation and publication counts.

**Table 2.** Top 50 Productive Journals for AI in Finance research.

Source	Documents	Citations	Source	Documents	Citations
Management Science	7	1019	Journal of Retailing and Consumer Services	6	91
Journal of Banking & Finance	7	793	European Financial Management	3	89
Technological Forecasting and Social Change	31	642	Journal of Forecasting	7	88
Finance Research Letters	54	499	Transportation Research Part E-Logistics and Transportation Review	7	86
Journal of Economic Dynamics & Control	3	389	International Review of Economics & Finance	14	79
Journal of Finance	2	362	Journal of the Operational Research Society	4	77
European Journal of Operational Research	12	353	Systems Research and Behavioral Science	7	74
Annual Review of Financial Economics, Vol 4	1	345	Information Systems Research	3	69
Journal of Hospitality Marketing & Management	4	336	Journal of Financial and Quantitative Analysis	2	66
International Journal of Contemporary Hospitality Management	8	274	European Journal of Finance	5	64
Journal of Business Research	11	261	MIS Quarterly	1	62
Research in International Business and Finance	20	235	International Journal of Emerging Markets	3	59
Journal of Behavioral and Experimental Finance	7	219	Quantitative Finance	11	59
International Journal of Accounting Information Systems	6	215	Computational Economics	14	54
California Management Review	1	200	European Business Organization Law Review	3	54
Energy Economics	27	185	Finance and Stochastics	1	46
Pacific-Basin Finance Journal	5	169	North American Journal of Economics and Finance	6	43
International Review of Financial Analysis	24	144	Journal of Business Finance & Accounting	1	42
Applications of Artificial Intelligence in Finance And Economics	10	141	Management Decision	5	40
Psychology & Marketing	4	136	International Review of Finance	2	39
Journal of Econometrics	5	133	Journal of the Academy of Marketing Science	2	37
Economic Research-Ekonomska Istrazivanja	6	130	E & M Ekonomie a Management	2	36
Journal of Marketing Research	2	129	Internet Research	3	35
Business Strategy and The Environment	6	122	Journal of Business Economics and Management	3	35
International Journal of Bank Marketing	2	121	International Transactions in Operational Research	2	33
Electronic Commerce Research and Applications	6	104	Energy Policy	1	32
Tourism Management	5	104	Financial Innovation	5	31
Economic Modelling	4	99	Journal of Research in Interactive Marketing	1	30
IEEE Transactions on Engineering Management	11	98	South African Journal of Economic and Management Sciences	1	28
Journal of Business Venturing	2	96	Applied Economics	5	27
Journal of Business & Industrial Marketing	5	92	Economic Analysis and Policy	5	27
Applied Economics Letters	5	91	Journal of Innovation & Knowledge	3	25

Source: Compiled by Authors using VoSviewer.

### 3.3 Top cited articles

To identify the most impactful contributions to this domain, we adopted a citation-based benchmarking approach, focusing on articles with at least 100 citations ( Bhukya & Paul, 2023). This analysis identified ten seminal articles, which are detailed in Table 3. With an average of 36.89 citations annually (CPY), a research article ( Das & Chen, 2007) published in Management Science has received 664 citations. The author ( Altman et al., 1994), in the Journal of Banking and Finance, gathered 464 citations (14.97 CPY) by offering a comparative evaluation of neural networks and traditional methods for corporate distress diagnosis, a foundational contribution to predictive analytics in finance. Several other studies have also made profound contributions to the Journal of Economic Dynamics and Control ( LeBaron et al., 1999) and received 386 citations (14.85 CPY) for their innovative exploration of time-series properties within artificial stock markets, bridging AI, and economic modelling. A nonparametric AI-driven method for pricing and hedging derivatives was presented ( Hutchinson et al., 1994) in The Journal of Finance, which received 355 citations (11.45 CPY). More recently, the authors ( Chod et al., 2020) highlighted the financial advantages of supply chain transparency and blockchain technology in Management Science, achieving 324 citations with an outstanding 64.80 CPY. The emerging significance of AI-driven decision making is further illustrated in the California Management Review ( Huang et al., 2019) (200 citations, 33.33 CPY) and Finance Research Letters ( Dowling & Lucey, 2023) (179 citations, 89.50 CPY). Despite being published only recently, the latter has demonstrated rapid scholarly engagement with AI in finance, emphasizing its critical role in shaping future research trajectories. The majority of these highly significant works were published in esteemed journals with ABDC classifications of A* or A, including Management Science, The Journal of Finance, and the Journal of Banking & Finance. Their large number of citations highlights a vital contribution to the growth of AI applications in the financial industry. This has laid the groundwork for future research. A thorough overview of these studies is given in Table 3, which also highlights the variety of approaches, research topics, and consequences that have influenced the academic environment of financial and AI studies. This emphasizes that AI is becoming a more significant factor in risk management, market analysis, and financial decision-making.

**Table 3.** Top 10 Frequently cited articles in AI and Finance Research.

Author	Year	Journal	Title	Years Since Publication	ABDC Ranking	Total Citations (TC)	TC per Year
Das, Sanjiv R.; Chen, Mike Y.	2007	Management Science	Yahoo! for Amazon: Sentiment Extraction from Small Talk on the Web	17	A*	664	36.89
Altman, Edward I.; Marco, Giancarlo; Varetto, Franco	1994	Journal of Banking & Finance	Corporate distress diagnosis: Comparisons using linear discriminant analysis and neural networks (the Italian experience)	30	A*	464	14.97
LeBaron, Blake; Arthur, W. Brian; Palmer, Richard	1999	Journal of Economic Dynamics and Control	Time series properties of an artificial stock market	25	A	386	14.85
Hutchinson, James M.; Lo, Andrew W.; Poggio, Tomaso	1994	The Journal of Finance	A Nonparametric Approach to Pricing and Hedging Derivative Securities Via Learning Networks	30	A*	355	11.45
Bisias, Dimitrios; Flood, Mark; Lo, Andrew W.; Valavanis, Stavros	2012	Annual Review of Financial Economics	A Survey of Systemic Risk Analytics	12	A	345	26.54
Chod, Jiri; Trichakis, Nikolaos; Tsoukalas, Gerry; Aspegren, Henry; Weber, Mark	2020	Management Science	On the Financing Benefits of Supply Chain Transparency and Blockchain Adoption	4	A*	324	64.80
Huang, Ming-Hui; Rust, Roland; Maksimovic, Vojislav	2019	California Management Review	The Feeling Economy: Managing in the Next Generation of Artificial Intelligence	5	A*	200	33.33
Lin, Hongxia; Chi, Oscar Hengxuan; Gursoy, Dogan	2020	Journal of Hospitality Marketing & Management	Antecedents of customers’ acceptance of artificially intelligent robotic device use in hospitality services	4	B	185	37.00
Huynh, Toan Luu Duc; Hille, Erik; Nasir, Muhammad Ali	2020	Technological Forecasting and Social Change	Diversification in the age of the 4th industrial revolution: The role of artificial intelligence, green bonds and cryptocurrencies	4	A	184	36.80
Dowling, Michael; Lucey, Brian	2023	Finance Research Letters	ChatGPT for (finance) research: The Bananarama conjecture	1	A	179	89.50

Source: Compiled by Authors using Bibliometrix.

### 3.4 Top productive authors

**Figure 3.**
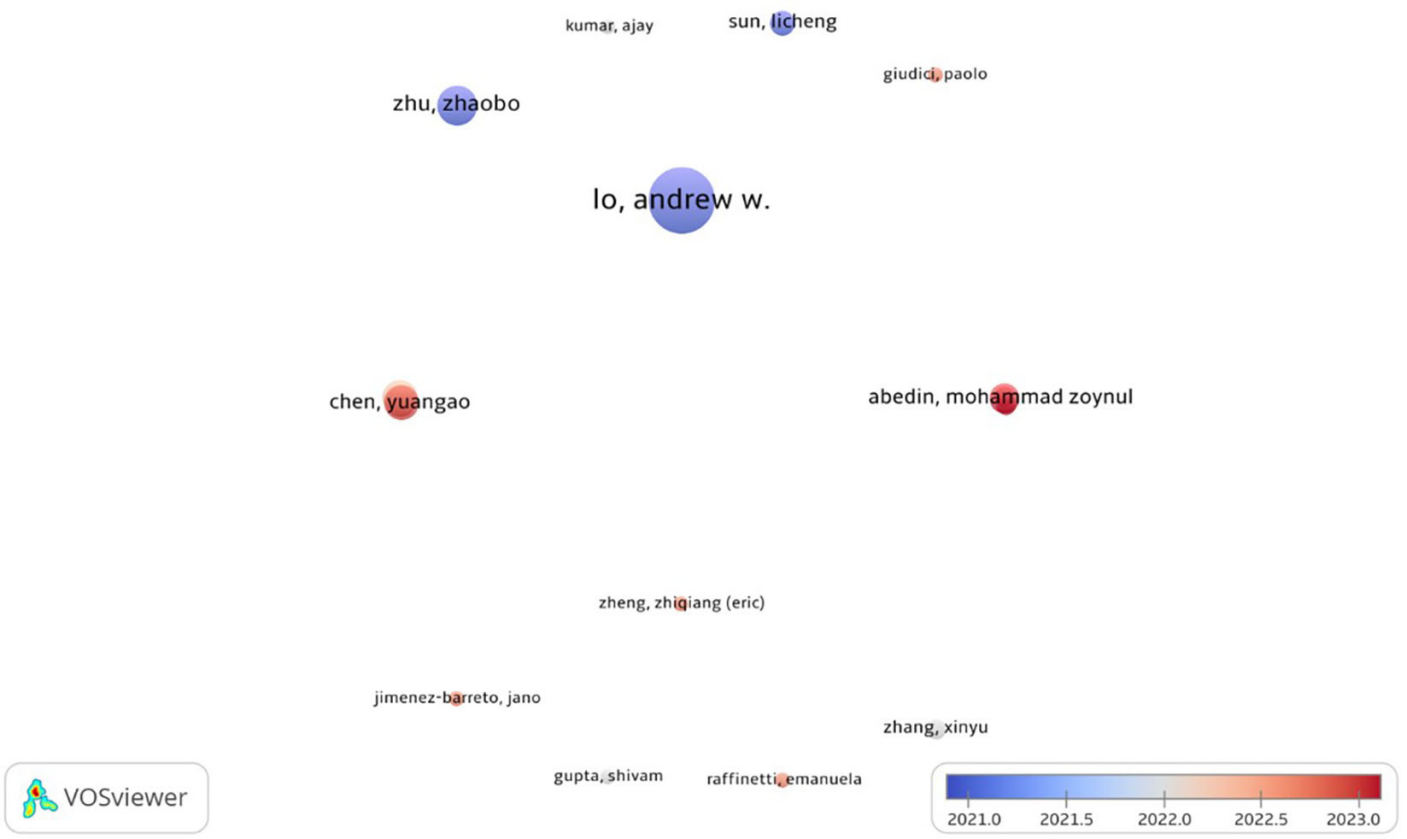
Author-network of top cited articles.

**Figure 4.**
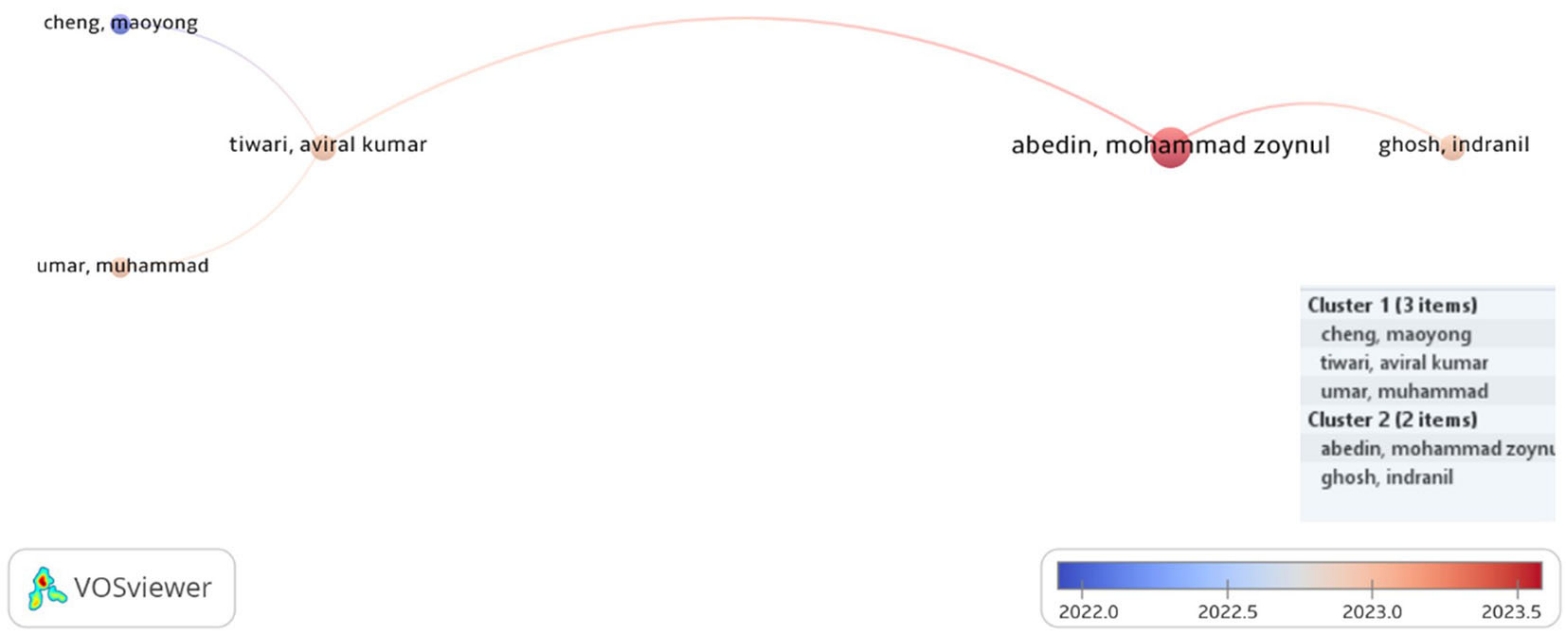
Productive and influential authors.

Our analysis revealed that 1966 writers contributed to the field of study. Nineteen played a significant role in developing the research area. Our aim was to identify the authors with the most cumulative citations and the most prolific authors. In terms of total citations (tc = 528) from 13 articles, Andrew W. Lo of the Massachusetts Institute of Technology stood out as the top contributor, highlighting his noteworthy influence in the field. With eight publications and 84 citations overall, Zhaobo Zhu dominated the list of authors with the most articles. Additional noteworthy contributors include Shasha Zhou and Yuangao Chen, each with seven publications and 211 and 176 cumulative citations, respectively, underscoring their strong influence in advancing academic discourse. Furthermore, researchers such as Maoyong Cheng (three publications, tc = 169) and Xingyang Lv (three publications, tc = 152) have also demonstrated focused and impactful research output. These contributions collectively reflect the dynamic and interdisciplinary efforts that drive the growth of AI and finance research. Figure 3, Figure 4, and Table 4 provide insightful snapshots of these leading scholars, emphasizing their pivotal roles in shaping the scholarly landscape.

**Table 4.** List of most Notable Authors.

Author	Documents	Citations
Andrew W.Lo	13	528
Shasha Zhou	7	211
Yuangao Chen	7	176
Maoyong Cheng	3	169
Xingyang Lv	3	152
Shuiqing Yang	6	150
Xinyu Zhang	4	124
Aviral Kumar Tiwari	4	118
Zhaobo Zhu	8	84
Jano Jimenez-Barreto	3	77
Zhiqiang (Eric) Zheng	3	69
Mohammad Zoynul Abedin	6	61
Paolo Giudici	3	60
Shivam Gupta	3	60
Ajay Kumar	3	60
Emanuela Raffinetti	3	60
Indranil Ghosh	4	55
Licheng Sun	5	51
Muhammad Umar	3	50

Source: Compiled by the Authors using VoSviewer.

### 3.5 Co-occurrence Network Analysis

**Figure 5.**
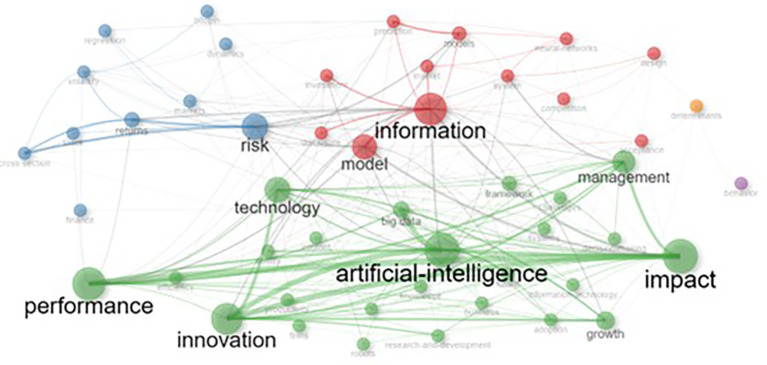
Co-occurrence network.

Figure 5 reveals the thematic structure of research on AI and Finance through a co-occurrence network in which distinct clusters of interconnected keywords are identified, each represented by a specific color. The largest cluster, highlighted in green, centers around “artificial intelligence” and includes closely linked terms like “machine learning,” “big data,” and “automation,” reflecting the widespread use of AI across diverse domains such as finance and technology. The red cluster emphasizes terms like “blockchain” and “cryptocurrency,” showcasing the amalgamation of AI with financial technologies, particularly in enhancing transparency, security, and digital innovation. The blue cluster focuses on “financial markets,” “risk assessment,” and “systemic analysis,” systemic analysis, demonstrating the use of AI in traditional financial research for predictive analytics and economic modelling. Additionally, smaller clusters, such as purple and orange clusters, capture niche areas such as behavioral analysis and decision-making systems. The multidisciplinary character of AI research is highlighted by these color-coded clusters, which integrate technological, financial, and economic development with computational advances. They also emphasized the crucial role that AI plays in tackling intricate problems in a variety of domains.

## 4. TCCM analysis

**Figure 6.**
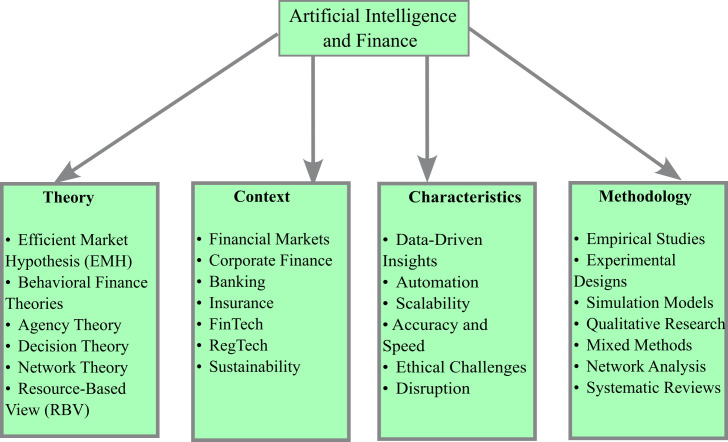
TCCM Framework for AI and Finance.

The TCCM approach improves the methodological arrangement and evaluation of multidisciplinary research on AI in finance. Four categories were created based on the results highlighted in Figure 6. The study first educates readers by carefully examining both the well-established and new hypotheses that support role of AI in changing financial systems. Second, there has been discussion and critical analysis of the settings in which the most important research on AI applications in finance is carried out. Third, the defining characteristics of AI that enable its diverse applications in finance are outlined, highlighting its impact on operations and strategies. Fourth, the research methodologies employed to investigate role of AI in finance are reviewed, highlighting the tools and approaches used for data collection and analysis.

### 4.1 Theoretical Foundations of AI in Finance

The analysis reveals six major hypotheses that explain how AI transforms financial innovation and decision making. The Efficient Market Hypothesis (EMH) explores how AI enhances the accuracy of financial market predictions and identifies inefficiencies that contribute to improved market efficiency ( Anshari et al., 2021; Buckley et al., 2021; Fama, 1970; Kurshan et al., 2021). Behavioral Finance Theories examine the influence of AI-driven sentiment analysis on investor psychology and decision-making, highlighting the psychological underpinnings of financial behavior ( Chartier et al., 2021). Agency Theory highlights AI’s potential to mitigate agency conflicts by enhancing transparency, monitoring, and corporate governance mechanisms, thereby reducing information asymmetry ( Jensen & Meckling, 1976; Kalkan, 2024). Decision theory examines how AI streamlines intricate financial decision-making procedures, especially in domains such as investment strategies and risk management ( Cao, 2023; Tversky & Kahneman, 1986). Network Theory illustrates AI’s significant role in systemic risk analysis, particularly through the modelling of financial networks, enabling a deeper understanding of interconnected risks ( Battiston et al., 2016). Finally, the Resource-Based View (RBV) positions AI as a strategic organizational resource that firms leverage to gain competitive advantages, innovate, and drive long-term growth ( Barney, 1991; Kitsios & Kamariotou, 2021; Machireddy et al., 2021). These theories offer a solid basis for comprehending the complex effects of AI on the financial industry to improve predictive analytics, decision-making procedures, and strategic management of financial institutions.

### 4.2 Contextual Foundations of AI in Finance

The literature on AI applications in finance spans diverse contexts, each contributing distinct insights into the transformative potential and the associated challenges of AI-driven innovation. Among these, the financial market context highlights AI’s application in algorithmic trading, market sentiment analysis, and fraud detection, showcasing its ability to enhance efficiency and security in trading environments ( Baldauf & Mollner, 2020; Cao, 2023). In Corporate Finance, AI is considered for financial forecasting, capital structure optimization, and strategic investment decisions, demonstrating its value in improving corporate decision-making processes ( Machireddy et al., 2021). The Banking sector leverages AI-powered solutions for credit risk assessment, customer relationship management, and the automation of loan approval processes, thereby enhancing operational efficiency and customer satisfaction ( Jain, 2023; Singh & Arora, 2023). Within Insurance, AI contributes to streamlining underwriting, managing claims, refining actuarial risk models, and fostering precision and cost efficiency ( Erem Ceylan, 2022; Rana et al., 2022; Zia & Kalia, 2022). The rise of FinTech showcases role of AI in developing innovative tools, such as robo-advisors, chatbots, and blockchain-driven systems, revolutionizing customer interactions, and transaction processes ( Kashyap, 2023). Finally, the Sustainability context explores AI’s contributions to environmental, social, and governance (ESG) investments, green finance, and carbon market analytics, highlighting its critical role in supporting sustainable economic development ( Hemanand et al., 2022; Macchiavello & Siri, 2022). These six contexts collectively demonstrate the breadth of AI’s impact on finance, operational efficiency, strategic decision-making, and sustainability initiatives.

### 4.3 Characteristics Underneath AI in Finance

AI’s transformative impact of AI on finance is underpinned by its unique technical, functional, and strategic characteristics, which enable it to address complex challenges and unlock new opportunities. A key component of AI in finance is Data-Driven Insights, which use big data to identify trends, facilitate predictive analytics, and improve decision-making in a variety of financial fields ( Go et al., 2020; Obschonka & Audretsch, 2020). Automation simplifies repetitive tasks, such as financial reporting, auditing, and trading, improves efficiency, and reduces operational burdens ( Jain, 2023; Singh & Arora, 2023). The Scalability of AI allows its application to expand seamlessly across various financial sectors and global markets, demonstrating its adaptability and potential ( Chen & Bellavitis, 2020; Jameaba, 2020). With Accuracy and Speed, AI delivers high-precision computations while accelerating complex financial processes, thereby ensuring timely and reliable outcomes ( Cao, 2020, 2023; Jameaba, 2020). However, the use of AI also brings ethical challenges such as concerns about bias, equity, and transparency in AI-powered systems, which call for strong governance structures ( Binns, 2018). AI also serves as a force for disruption, transforming traditional financial paradigms, fostering innovation, and driving new market dynamics ( Kalkan, 2024). Together, these characteristics displays capacity of AI to enhance efficiency, reduce costs, and redefine strategies, making it a pivotal driver of financial innovation and evolution.

### 4.4 Methodological Foundations of AI in Finance

AI research within finance domain has adopted a range of methodological approaches to investigate its multifaceted implications and applications. Empirical Studies show that machine learning algorithms perform predictive modelling and financial analysis, offering data-driven insights into market behavior and trends ( Cao, 2023). Experimental Designs focus on testing AI-based solutions for critical functions, such as fraud detection, trading strategies, and risk management, enabling practical evaluations of effectiveness of AI ( Kashyap, 2023). Simulation Models create artificial markets and scenarios, thus providing a framework for assessing systemic risks and market dynamics under controlled conditions ( Battiston et al., 2016). Qualitative Research delves into stakeholder perceptions and attitudes toward AI adoption in finance, shedding light on behavioral and organizational factors ( Ridzuan et al., 2024). Mixed Methods integrate quantitative and qualitative approaches, offering a holistic perspective of impact and potential of AI ( Kitsios & Kamariotou, 2021; Machireddy et al., 2021; Tang, 2021). Network Analysis employs AI-driven graph analytics to map financial systems and uncover relationships, thereby enhancing the systemic understanding ( Jain, 2023; Singh & Arora, 2023). Additionally, Systematic Reviews synthesize existing literature to identify emerging trends and research gaps, guiding future inquiries ( Baldauf & Mollner, 2020; Cao, 2023). Despite considerable advancements, there are still gaps in the use of cutting-edge approaches to better grasp the revolutionary role of AI in finance, such as agent-based modelling and neuroscience ( Go et al., 2020).

## 5. Research Gaps and Future Directions

The TCCM framework identifies significant research gaps and proposes avenues for future exploration to enhance the understanding of AI’s role in finance. Theoretically, there is scope to integrate emerging frameworks, such as technological determinism and the adaptive markets hypothesis, to deepen insights into AI’s influence on financial decision-making ( Kalkan, 2024). Contextually, applications of AI in microfinance, rural banking, and emerging economies remain underexplored, presenting opportunities to address unique challenges in underserved markets ( Hemanand et al., 2022; Macchiavello & Siri, 2022). Ethical concerns, such as fairness, transparency, and explainability in AI-driven algorithms, require urgent attention to ensure accountability and alignment with ethical standards ( Binns, 2018). Methodologically, adopting innovative approaches, including neuroscience and behavioral experiments, can provide novel insights into the cognitive and psychological dimensions of financial applications of AI ( Go et al., 2020; Obschonka & Audretsch, 2020). Additionally, interdisciplinary research exploring intersection of AI with domains such as climate finance, blockchain, and global economic resilience can further expand its applications and address broader challenges ( Barney, 1991; Kitsios & Kamariotou, 2021; Machireddy et al., 2021).

From a country perspective ( Table 5), China leads research output with 337 documents, while the USA demonstrates the highest research impact with 4628 citations from 128 documents, highlighting its dominance in high-impact studies. England, with 75 documents and 1137 citations, also showed a significant influence. Among European nations, Italy (33 documents, 1082 citations) and France (55 documents, 675 citations) make notable contributions. In Asia, China stands out, followed by Taiwan (35 documents, 460 citations), and India (21 documents, 419 citations), underlining the region’s growing focus on AI-driven financial innovation. Smaller yet impactful contributions came from Vietnam (13 documents, 536 citations) and Malaysia (19 documents, 308 citations). Australia (34 documents, 515 citations) and Germany (29 documents, 535 citations) represent consistent efforts toward AI finance research. Emerging players such as South Africa (6 documents, 318 citations) and the UAE (7 documents, 312 citations) also highlight their involvement, albeit with fewer publications.

**Table 5.** Most cited countries for AI and Finance Research.

Country	Documents	Citations
USA	128	4628
CHINA	337	3507
ENGLAND	75	1137
ITALY	33	1082
FRANCE	55	675
VIETNAM	13	536
GERMANY	29	535
AUSTRALIA	34	515
TAIWAN	35	460
INDIA	21	419
SOUTH AFRICA	6	318
UAE	7	312
MALAYSIA	19	308
SPAIN	21	242
IRELAND	11	234

Source: Compiled by Authors using VoSviewer.

**Figure 7.**
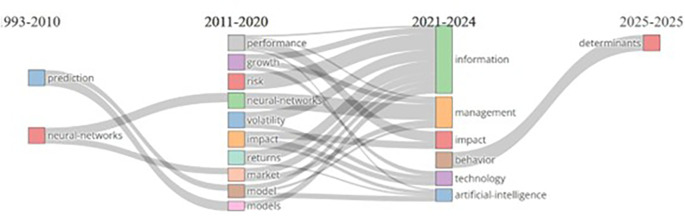
Thematic evolution of AI and Finance Research literature.

The Sankey diagram ( Figure 7) visualizes the evolution of AI and finance research themes over distinct periods, illustrating the dynamic progression of key topics. From 1993 to 2010, the focus was on prediction and neural networks, with predictive modelling in finance and the growing application of neural networks in decision-making. During 2011–2020, research diversified, with neural networks expanding into areas such as risk, volatility, and market studies. Themes such as growth, impact, and returns emerged, reflecting broader applications in financial performance and economic growth. The period from 2021 to 2024 marked a shift toward AI and technology, with an increased emphasis on the practical and managerial aspects of research alongside themes of information and management. The recent emergence of behavior as a key theme suggests a focus on decision making, consumer behavior, and organizational psychology.

Looking ahead to 2025, the projected focus on determinants signals a growing interest in understanding the underlying factors that drive success or failure in various domains. While the rise of impact, information, and management indicates a move toward practical applications, themes such as neural networks remain consistent over time, reflecting their central role in the increase of AI and finance research. The future focus on determinants points to a trend toward deeper causal analysis to understand the factors shaping impact of AI on finance.

**Figure 8.**
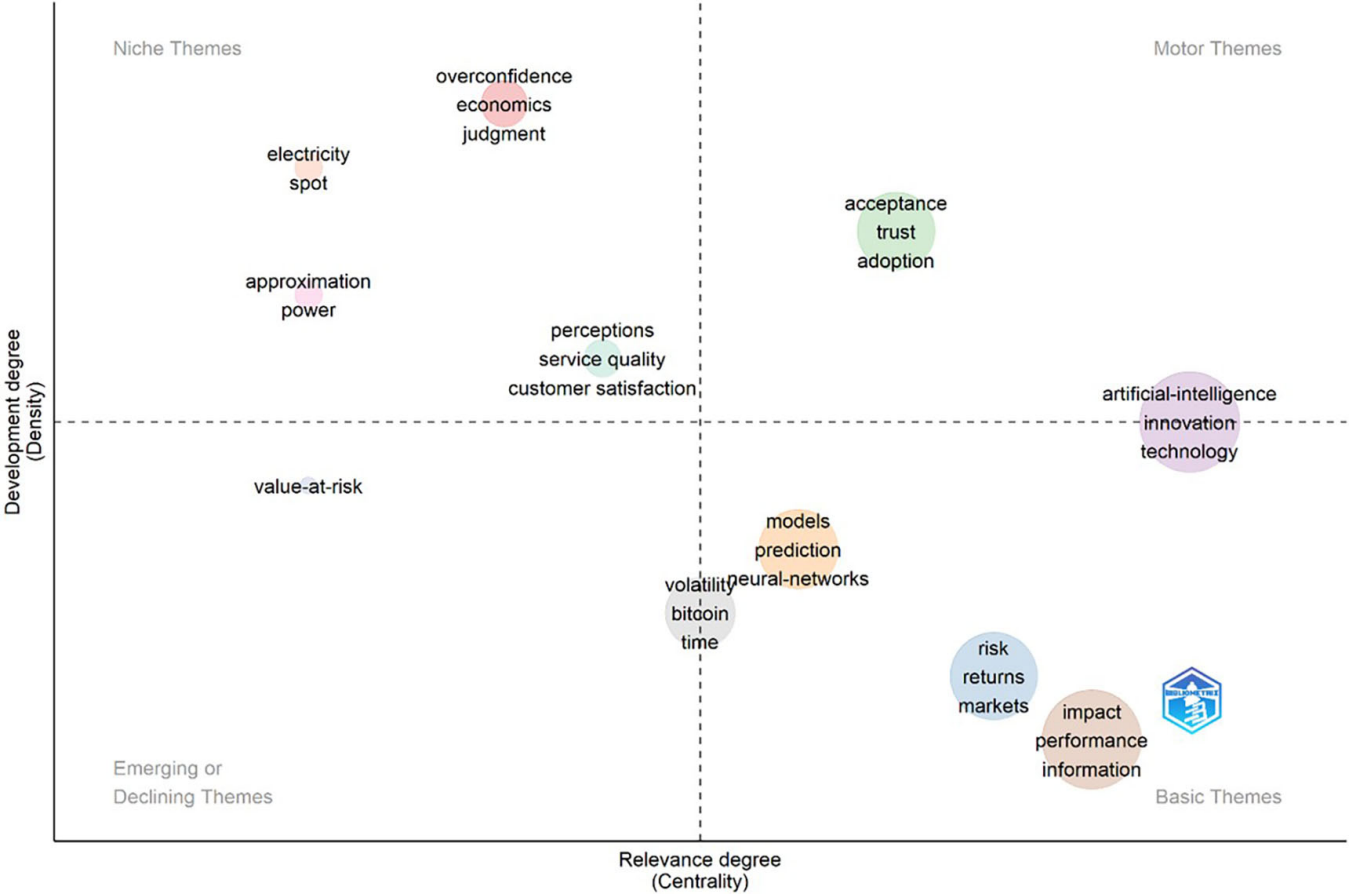
Thematic Map on Conceptual Structure of AI and Finance Research.

The research subjects in the fields of AI and finance are given by a thematic map ( Figure 8), which groups them according to two criteria: density (development degree) and centrality (relevance degree). Each of the four quadrants constituting the map represents a distinct topic. The high centrality and density of the motor topics (top-right quadrant) indicate well-developed topics that are essential to the field of study. Key topics in this quadrant include AI, Innovation, and Technology, which are pivotal for driving advancements within the domain. Themes with high density but low centrality are seen in the Niche Themes (top-left quadrant), signifying specialized fields with minimal ties to the larger research environment.

Topics such as Overconfidence, Economics, Judgment, and Electricity, Spot Markets fall into this category, representing niche applications and behavioral finance aspects. The undeveloped Emerging or Declining Themes (bottom-left quadrant), such as value-at-risk, have low centrality and density, which indicates that current research has shown less interest in these subjects. Lastly, the bottom-right quadrant’s Basic Themes, which have high centrality but low density, stand for fundamental ideas such as risk, returns, markets, impact, performance, and information. These ideas are essential for financial analysis and investment studies, but they might need more work to realize their full potential.

### 5.1 Contextual Future Research Directions in AI and Finance

The industry has seen a dramatic change as a result of the rapid development of AI, which has opened new avenues for risk management, predictive analysis, and more effective decision-making. The use of AI in financial operations is still developing, and a number of important areas require further research. As technology advances and the financial landscape changes, more research is necessary to fully understand how AI propels innovation in markets and financial institutions. A promising path toward enhancing operational effectiveness and financial stability is provided by the AI’s capacity to handle enormous amounts of data and deliver insights into dynamic environments. With increasing influence of AI, particularly in areas such as algorithmic trading, fraud detection, and financial forecasting, the following future research questions (FRQs) can guide further investigation into the role of AI in finance.

**FRQ1:** *How can AI-driven predictive models enhance decision-making in volatile financial markets, particularly during economic downturns or crises?*

**FRQ2:** *How does the use of AI to financial decision-making raise ethical questions, particularly with regard to accountability, transparency, and fairness?*

**FRQ3:** *What effects will AI deployment have on conventional risk management techniques and the part that human decision-makers play in these procedures in financial institutions?*

**FRQ4:** *How can AI innovations improve financial inclusion, particularly in underserved or emerging markets?*

**FRQ5:** *What are the longstanding effects of AI on investor behaviour and financial market dynamics, particularly regarding the automation of trading and advisory services?*

**FRQ6:** *Can AI play a role in enhancing sustainability in finance by supporting green finance initiatives and responsible investment practices?*

### 5.2 Characteristics Future Research Directions in AI and Finance

AI in finance is driven not only by its technical capabilities but also by its potential to disrupt traditional financial models and improve efficiency. Future research can explore the interplay between AI technologies and behavioral factors that influence decision-making processes in finance. Key areas of interest include the following.

**FRQ1:** How does AI-driven sentiment analysis affect investor psychology and market movements, particularly in volatile or uncertain periods?

**FRQ2:** What role does AI play in reshaping financial advisory services, and how do consumers perceive the trustworthiness and effectiveness of AI-driven advice compared to traditional human advisors?

**FRQ3:** How can AI be leveraged to improve decision-making under uncertainty, particularly in complex financial markets where human cognition may be limited?

As AI technology continues to evolve, its interaction with human behavior and cognitive processes in finance remains a critical area for future research. Understanding how AI enhances or challenges human decision-making and the ethical implications of these changes is crucial for shaping the future of finance. Research in this area should consider the potential of AI to not only optimize financial outcomes but also its role in influencing consumer behavior and financial practices.

### 5.3 Methodological Future Research Directions

AI is used in a large range of financial products, from algorithmic trading to fraud detection, risk management, and financial forecasting. Quantitative methods, such as statistical analysis and machine learning models, are the mainstays of current research in this field. Model overfitting, data bias, and limited interpretability of sophisticated AI systems are some of the methodological problems that still exist. Future research can address these issues by implementing a greater variety of creative techniques to increase the precision, dependability, and interpretability of AI-driven financial models. Using text analytics and natural language processing (NLP) tools to examine vast volumes of financial data from many sources, such as business filings, social media, and financial news, is a possible path for methodological progress. Using named entity identification, topic modelling, and sentiment analysis, researchers can learn more about investor behavior, market mood, and patterns that affect financial decision making. These methods can provide more accurate predictions of market movements and risk factors while also addressing biases inherent in structured financial data.

Additionally, simulation methodologies such as agent-based modelling and fuzzy cognitive mapping offer promising approaches for understanding complex interactions in financial systems. These techniques allow for the modelling of dynamic financial environments where individual agents, such as investors or firms, interact under uncertain conditions. Research on how AI affects financial stability, risk propagation, and market behavior can be conducted using agent-based models. On the other hand, fuzzy cognitive mapping can offer a better understanding of systemic risk and role of AI in financial crises by visualizing and comprehending the interaction between AI-driven decisions and other macroeconomic factors. Lastly, qualitative approaches can be significant in comprehending the behavioral consequences of AI adoption, as AI continues to upend conventional banking practices. Stakeholder opinions about the use of AI in finance can be investigated using case studies, expert interviews, and interviews. The impact of AI on decision making, organizational behavior, and the future of financial advice services could be the main topics of discussion. Combining these qualitative insights with quantitative data can offer a holistic view of transformative potential of AI in finance.

## 6. Implications

### 6.1 Managerial Implications

Our research highlights the managerial implications for financial institutions and other stakeholders in the finance sector. Financial institutions should be mindful of AI’s potential to influence customer behavior in both beneficial and detrimental ways. While AI can drive efficiency and improve decision-making, its use in customer-facing applications must be approached with caution to avoid ethical concerns such as algorithmic bias or lack of transparency. Marketers, for example, should focus on promoting AI-based financial tools in ways that are transparent and aligned with consumers’ best interests, rather than relying solely on automation for decision-making.

### 6.2 Implications for Policymakers

Policymakers need to address the ethical and legal issues raised by AI in finance. It is imperative that governments and authorities put in place mechanisms that guarantee accountability, transparency, and justice in AI algorithms, given the widespread use of AI in decision-making. Oversight is required as AI systems in finance have become more sophisticated to prevent market manipulation, discrimination, and data privacy violations. Furthermore, regulators should focus on creating standards for AI literacy among consumers to help them understand the implications of AI-driven financial products/services.

### 6.3 Ethical Implications

AI in finance has several significant ethical implications. Although AI systems can improve decision making, they can also reinforce pre-existing biases, raising concerns about equality and fairness. Ethics pertaining to the independence of AI-driven judgments, openness in algorithmic procedures, and protection of consumer rights must be addressed as AI continues to have an impact on the financial industry. Researchers and practitioners should pay close attention to these issues, ensuring that AI adoption in finance does not disproportionately affect vulnerable populations or widen existing financial disparities.

## 7. Conclusion

This study has some limitations. First, while we have focused on impact of AI in finance, the rapidly evolving nature of AI technologies suggests that new research is needed to account for emerging trends and applications. Additionally, most of the reviewed studies focus on Western financial markets. This could restrict the extent to which our results can be applied in other areas. Future studies should examine the cross-cultural uses of AI in finance to determine how various markets react to AI-driven financial tools. Finally, although we have outlined the main ethical issues, further study is required to fully understand the long-term societal effects of AI in finance, especially regarding privacy, surveillance, and consumer autonomy. AI holds the prospect of transforming the financial sector by enhancing decision making, minimizing risks, and improving operational efficiency. However, as emphasized in our research, its implementation brings certain challenges, including ethical concerns, managerial complexities, and policy implications, all of which require responsible attention.

## Data Availability

Zenodo: AI and Finance, https://doi.org/10.5281/zenodo.14410427 ( Singh, P. K, 2024). This project contains the following underlying data:
1.
AI and Fin.bib
2. AI fin.txt License: Data is available under Creative Commons Attribution 4.0 International

## References

[ref1] Agrawal A Gans J Goldfarb A : *Prediction Machines: The Simple Economics of Artificial Intelligence.* Harvard Business Review Press;2018.

[ref2] Altman EI Marco G Varetto F : Corporate distress diagnosis: Comparisons using linear discriminant analysis and neural networks (the Italian experience). *J. Bank. Financ.* 1994;18(3):505–529. 10.1016/0378-4266(94)90007-8

[ref3] Anshari M Almunawar MN Masri M : Financial Technology with AI-Enabled and Ethical Challenges. *Society.* 2021;58(3):189–195. 10.1007/s12115-021-00592-w

[ref4] Aria M Cuccurullo C : Bibliometrix: An R-tool for comprehensive science mapping analysis. *J. Informet.* 2017;11(4):959–975. 10.1016/j.joi.2017.08.007

[ref5] Arner DW Barberis J Buckley RP : FinTech, RegTech, and the Reconceptualization of Financial Regulation. *Northwest. J. Int. Law Bus.* 2016;37(3):371–413.

[ref6] Baldauf M Mollner J : High-frequency trading and market performance. *J. Financ.* 2020;75(3):1495–1526. 10.1111/jofi.12875

[ref7] Barney J : Firm resources and sustained competitive advantage. *J. Manag.* 1991;17(1):99–120. 10.1177/014920639101700108

[ref8] Battiston S Caldarelli G May RM : The price of complexity in financial networks. *Proc. Natl. Acad. Sci.* 2016;113(36):10031–10036. 10.1073/pnas.1521573113 27555583 PMC5018742

[ref9] Bhukya R Paul J : Social influence research in consumer behavior: What we learned and what we need to learn?–A hybrid systematic literature review. *J. Bus. Res.* 2023;162:113870. 10.1016/j.jbusres.2023.113870 Reference Source

[ref10] Binns R : Fairness in machine learning: Lessons from political philosophy. 2018;149–159.

[ref11] Bock DE Wolter JS Ferrell OC : Artificial intelligence: Disrupting what we know about services. *J. Serv. Mark.* 2020;34(3):317–334. 10.1108/JSM-01-2019-0047/full/html

[ref12] Brynjolfsson E McAfee A : *The Second Machine Age: Work, Progress, and Prosperity in a Time of Brilliant Technologies.* W. W. Norton & Company;2014.

[ref13] Buckley RP Zetzsche DA Arner DW : Regulating artificial intelligence in finance: Putting the human in the loop. *Syd. Law Rev.* 2021;43(1):43–81. 10.3316/informit.676004215873948

[ref14] Cao L : AI in finance: A review. *Available at SSRN 3647625.* 2020. Reference Source

[ref15] Cao L : AI in Finance: Challenges, Techniques, and Opportunities. *ACM Comput. Surv.* 2023;55(3):1–38. 10.1145/3502289

[ref16] Chartier E Bowden I Pinkerton M : Behavioral finance: the impact of artificial intelligence and social media analytics. *Available at SSRN 3794039.* 2021. Reference Source

[ref17] Chen Y Bellavitis C : Blockchain disruption and decentralized finance: The rise of decentralized business models. *J. Bus. Ventur. Insights.* 2020;13:e00151. 10.1016/j.jbvi.2019.e00151 Reference Source

[ref18] Chod J Trichakis N Tsoukalas G : On the Financing Benefits of Supply Chain Transparency and Blockchain Adoption. *Manag. Sci.* 2020;66(10):4378–4396. 10.1287/mnsc.2019.3434

[ref19] Chong TT Lee K Tan HB : Artificial Intelligence and its applications in finance: An overview. *J. Financ. Technol.* 2017;1(1):18–25.

[ref20] Coglianese C Lehr D : Transparency and algorithmic governance. *Adm. Law Rev.* 2019;71(1):1–56. Reference Source

[ref21] Das SR Chen MY : Yahoo! for Amazon: Sentiment Extraction from Small Talk on the Web. *Manag. Sci.* 2007;53(9):1375–1388. 10.1287/mnsc.1070.0704

[ref22] Davenport T Ronanki R : Artificial Intelligence for the Real World. *Harv. Bus. Rev.* 2018;96(1):108–116.

[ref23] Dhamija P Bag S : Role of artificial intelligence in operations environment: a review and bibliometric analysis. *TQM J.* 2020;32(4):869–896. 10.1108/TQM-10-2019-0243

[ref24] Dixon MF Halperin I Bilokon P : *Machine Learning in Finance: From Theory to Practice.* Springer International Publishing;2020. 10.1007/978-3-030-41068-1

[ref25] Dowling M Lucey B : ChatGPT for (finance) research: The Bananarama conjecture. *Financ. Res. Lett.* 2023;53:103662. 10.1016/j.frl.2023.103662 Reference Source

[ref26] Engles E Bolze JD : Artificial intelligence based service implementation. 2020. Reference Source

[ref27] Erem Ceylan I : The Effects of Artificial Intelligence on the Insurance Sector: Emergence, Applications, Challenges, and Opportunities. Bozkuş Kahyaoğlu S , editor. *The Impact of Artificial Intelligence on Governance, Economics and Finance.* Singapore: Springer Nature;2022;2: pp.225–241. 10.1007/978-981-16-8997-0_13

[ref28] Fama EF : Efficient capital markets: A review of theory and empirical work. *J. Financ.* 1970;25(2):383–417. 10.2307/2325486

[ref29] Go EJ Moon J Kim J : Analysis of the current and future of the artificial intelligence in financial industry with big data techniques. *Glob. Bus. Finance Rev.* 2020;25(1):102–117. 10.17549/gbfr.2020.25.1.102 Reference Source

[ref30] Hemanand D Mishra N Premalatha G : Applications of intelligent model to analyze the green finance for environmental development in the context of artificial intelligence. *Comput. Intell. Neurosci.* 2022;2022(1):2977824. 10.1155/2022/2977824 35845917 PMC9283008

[ref31] Henrique BM Sobreiro VA Kimura H : Literature review: Machine learning techniques applied to financial market prediction. *Expert Syst. Appl.* 2019;124:226–251. 10.1016/j.eswa.2019.01.012 Reference Source

[ref32] Huang M-H Rust R Maksimovic V : The Feeling Economy: Managing in the Next Generation of Artificial Intelligence (AI). *Calif. Manag. Rev.* 2019;61(4):43–65. 10.1177/0008125619863436

[ref33] Hutchinson JM Lo AW Poggio T : A Nonparametric Approach to Pricing and Hedging Derivative Securities Via Learning Networks. *J. Financ.* 1994;49(3):851–889. 10.1111/j.1540-6261.1994.tb00081.x

[ref34] Ingale KK Paluri RA : Financial literacy and financial behaviour: a bibliometric analysis. *Rev. Behav. Finance.* 2020;14(1):130–154. 10.1108/RBF-06-2020-0141

[ref35] Jain R : Role of artificial intelligence in banking and finance. *J. Manag. Sci.* 2023;13(3):1–4. 10.26524/jms.13.27 Reference Source

[ref36] Jameaba M-S : Digitization Revolution, FinTech Disruption, and Financial stability: Using the Case of Indonesian Banking Ecosystem to highlight wide-ranging digitization opportunities and major challenges. IO: Productivity. 2020. Reference Source

[ref37] Jensen MC Meckling WH : Theory of the firm: Managerial behavior, agency costs, and ownership structure. *J. Financ. Econ.* 1976;3(4):305–360. 10.1016/0304-405X(76)90026-X

[ref38] Kalkan G : The Impact of Artificial Intelligence on Corporate Governance. *Корпоративные Финансы.* 2024;18(2):17–25. 10.17323/j.jcfr.2073-0438.18.2.2024.17-25 Reference Source

[ref39] Kashyap R : DeFi Security: Turning The Weakest Link Into The Strongest Attraction. *arXiv. 10.48550/arXiv.2312.00033.* 2023.

[ref40] Kitsios F Kamariotou M : Artificial intelligence and business strategy towards digital transformation: A research agenda. *Sustainability (Switzerland).* 2021;13(4):1–16. 10.3390/su13042025

[ref41] Kurshan E Chen J Storchan V : On the current and emerging challenges of developing fair and ethical AI solutions in financial services. *ICAIF’21: 2nd ACM International Conference on AI in Finance.* 2021; pp.1–8. 10.1145/3490354.3494408

[ref42] LeBaron B Arthur WB Palmer R : Time series properties of an artificial stock market. *J. Econ. Dyn. Control.* 1999;23(9):1487–1516. 10.1016/S0165-1889(98)00081-5

[ref43] Li Z Zhang W : Technology in education: Addressing legal and governance challenges in the digital era. *Educ. Inf. Technol.* 2024;1–31. Reference Source

[ref44] Macchiavello E Siri M : Sustainable finance and fintech: Can technology contribute to achieving environmental goals? A preliminary assessment of ‘green fintech’and ‘sustainable digital finance. *Eur. Co. Financ. Law Rev.* 2022;19(1):128–174. 10.1515/ecfr-2022-0005/html

[ref45] Machireddy JR Rachakatla SK Ravichandran P : Leveraging AI and Machine Learning for Data-Driven Business Strategy: A Comprehensive Framework for Analytics Integration. *Afr. J. Artif. Intell. Sustain. Dev.* 2021;1(2):127–150.

[ref46] McCarthy J Minsky M Rochester N : A proposal for the Dartmouth summer research project on artificial intelligence. 1956.

[ref47] Mohamed A Najafabadi MK Wah YB : The state of the art and taxonomy of big data analytics: view from new big data framework. *Artif. Intell. Rev.* 2020;53(2):989–1037. 10.1007/s10462-019-09685-9

[ref48] Nabipour M Nayyeri P Jabani H : Predicting stock market trends using machine learning and deep learning algorithms via continuous and binary data; a comparative analysis. *IEEE Access.* 2020;8:150199–150212. 10.1109/ACCESS.2020.3015966 Reference Source

[ref49] Obschonka M Audretsch DB : Artificial intelligence and big data in entrepreneurship: a new era has begun. *Small Bus. Econ.* 2020;55(3):529–539. 10.1007/s11187-019-00202-4

[ref50] Pal A Tiwari CK Behl A : Blockchain technology in financial services: a comprehensive review of the literature. *J. Glob. Oper. Strateg. Sourc.* 2021;14(1):61–80. 10.1108/JGOSS-07-2020-0039/full/html

[ref51] Patil D Rane NL Desai P : Machine learning and deep learning: Methods, techniques, applications, challenges, and future research opportunities. *Trustworthy Artificial Intelligence in Industry and Society.* 2024; pp.28–81. 10.70593/978-81-981367-4-9_2 Reference Source

[ref52] Paul J Rosado-Serrano A : Gradual internationalization vs born-global/international new venture models. *Int. Mark. Rev.* 2019;36(6):830–858. 10.1108/IMR-10-2018-0280

[ref53] Rana A Bansal R Gupta M : Emerging Technologies of Big Data in the Insurance Market. Sood K Dhanaraj RK Balusamy B , editors. *Big Data: A Game Changer for Insurance Industry.* Emerald Publishing Limited;2022; pp.15–34. 10.1108/978-1-80262-605-620221002/full/html

[ref54] Ridzuan NN Masri M Anshari M : AI in the Financial Sector: The Line between Innovation, Regulation and Ethical Responsibility. *Information.* 2024;15(8):432. 10.3390/info15080432 Reference Source

[ref55] Russell S Norvig P : *Artificial Intelligence: A Modern Approach.* Pearson Education;2016.

[ref56] Shin DDH : Socio-technical design of algorithms: Fairness, accountability, and transparency. 2019. Reference Source

[ref62] Singh PK : AI and Finance.[Data set]. *Zenodo.* 2024. 10.5281/zenodo.14410427

[ref57] Singh H Arora S : Artificial Intelligence & Machine Learning Models for Credit Scoring and Risk Management. *IITM J. Manag. IT.* 2023;14(1 and 2):7–13. Reference Source

[ref58] Taeihagh A : Governance of artificial intelligence. *Polic. Soc.* 2021;40(2):137–157. 10.1080/14494035.2021.1928377 Reference Source

[ref59] Tang Y : Corporate finance management in the age of ARTIFICIAL intelligence. *Int. J. Front. sociol.* 2021;3(12):141–146. Reference Source

[ref60] Tversky A Kahneman D : Rational choice and the framing of decisions. *J. Bus.* 1986;59(4):S251–S278. 10.1086/296365

[ref61] Zia A Kalia P : Emerging technologies in insurance sector: evidence from scientific literature. *Big Data: A Game Changer for Insurance Industry.* 2022; pp.43–63. 10.1108/978-1-80262-605-620221004/full/html

