# Supplementary material for: Artificial Intelligence and Finance: A bibliometric review on the Trends, Influences, and Research Directions
Source: F1000Res. 2025 Jan 23;14:122. [Version 1] doi: 10.12688/f1000research.160959.1 (PMC11795023; doi:10.12688/f1000research.160959.1)
Supplement: Peer review history

# Reviewer report: Reviewer response for version 1

**DOI:** 10.5256/f1000research.176927.r362855

**Published:** 2025-02-04

**Authors:** Rajeev Semwal

**Affiliations:** 1 Amity University, Noida, India

**1. Is the work clearly and accurately presented and does it cite the current literature?**

**Response:** **Yes**

**Explanation:** The paper is well-structured and provides a comprehensive review of the intersection between artificial intelligence (AI) and finance. The presentation follows a logical flow, beginning with an introduction to AI's impact on finance, a review of relevant literature, a detailed discussion of the bibliometric methodology, and a structured analysis using the TCCM framework. The paper effectively conveys its objectives, research questions, and findings without excessive complexity. The citations include a mix of foundational theories (e.g., Efficient Market Hypothesis, Decision Theory) and contemporary research, ensuring that the literature review remains current and relevant. The use of bibliometric tools such as *VOSviewer* and *Bibliometrix* further strengthens the credibility of the study, providing a systematic analysis of publication trends, key authors, and influential works.

**Recommendation:**

 The clarity of the paper is commendable, and no major revisions are needed in terms of structure or citation quality. However, the introduction could benefit from a more concise summary of the key bibliometric trends identified, helping readers quickly grasp the significance of the study. Additionally, while the literature cited is extensive, the authors could briefly acknowledge any limitations related to potential publication bias, particularly the exclusion of non-English and non- *Web of Science* indexed studies.

**2. Is the study design appropriate and is the work technically sound?**

**Response:** **Yes**

**Explanation:** The study employs a robust bibliometric approach, which is a widely accepted method for identifying research trends and knowledge gaps in a given domain. The authors use a well-defined process for data collection, screening, and analysis, ensuring methodological rigor. The adoption of the *TCCM framework* provides a structured way to categorize existing research based on theoretical perspectives, contextual applications, methodological approaches, and key characteristics. This framework enhances the study’s depth by not only mapping existing literature but also proposing future research directions. The reliance on the *Web of Science* (WoS) database ensures access to high-impact, peer-reviewed publications, lending credibility to the findings. The use of bibliometric tools ( *Bibliometrix* and *VOSviewer*) for co-citation analysis, keyword mapping, and research impact assessment adds further technical validity.

**Recommendation:** While the study design is appropriate, the authors could briefly discuss any potential biases associated with bibliometric analysis. For example, the exclusion of conference papers and preprints could mean that some emerging research trends are not captured. Additionally, acknowledging any limitations related to the reliance on citation metrics (which can be influenced by journal impact factors rather than intrinsic research quality) would enhance transparency.

**3. Are sufficient details of methods and analysis provided to allow replication by others?**

**Response:** **Partly**

**Explanation:** The paper outlines the methodology in a structured manner, detailing the data collection process, the use of specific bibliometric tools, and the screening process for relevant articles. The inclusion of a three-step selection process (identification, screening, and final inclusion) ensures clarity in how studies were chosen. However, some critical details are missing that could impact replication. For instance, the exact parameters used in *VOSviewer* for network visualization (e.g., clustering thresholds, link strength criteria) are not mentioned. Similarly, while the *TCCM framework* is referenced, it is unclear how the classification of studies into its four components was conducted—was it manually coded, or was a software-assisted text analysis used?

**Recommendation:** The authors should provide additional methodological details to enhance replicability. Specifically, they should:

- Include a more detailed description of how bibliometric data was cleaned and processed.
- Specify the exact search terms and filters used in *Web of Science*.

**4. If applicable, is the statistical analysis and its interpretation appropriate?**

**Response:** **Yes**

**Explanation:** The statistical analysis used in the paper is appropriate for a bibliometric review. The study effectively utilizes co-citation networks, citation count analysis, and research trend evaluations to identify influential works and key research domains. The thematic evolution analysis, which traces how AI and finance research has developed over time, is particularly useful in highlighting emerging research trends. The authors also provide a Sankey diagram to illustrate shifts in research focus, adding a strong visual representation of the field’s progression. The citation-based benchmarking approach used to identify high-impact studies is an established method in bibliometrics, and the interpretation of these results aligns well with broader trends in AI and finance.

**Recommendation:** No major revisions are required for the statistical analysis. However, the authors could improve the discussion by briefly addressing potential limitations of citation-based impact metrics. For example, some highly cited papers might be review articles rather than empirical studies, and self-citations could also influence citation counts. Including a short paragraph on such limitations would add nuance to the discussion.

**5. Are all the source data underlying the results available to ensure full reproducibility?**

 Response: Yes

**Explanation:**

 The study ensures transparency by making its dataset publicly available on Zenodo, with a provided DOI link. This allows other researchers to access the raw bibliometric data used in the analysis, facilitating reproducibility. The inclusion of citation details, thematic mappings, and network visualizations further supports the integrity of the study.

 Additionally, the authors utilize widely accepted bibliometric tools (VOSviewer and Bibliometrix) for analysis, making it easier for others to replicate their methodology. The structured approach to data selection, processing, and screening ensures that all source materials are well-documented.

**Recommendation:**

 While data accessibility is well-addressed, the authors could enhance reproducibility by Providing a step-by-step guide or supplementary material outlining how to replicate the analysis using the shared dataset. Clarifying whether any pre-processing scripts or software configurations (e.g., parameter settings in VOSviewer) are included in the repository.

**6. Are the conclusions drawn adequately supported by the results?**

**Response:** **Partly**

**Explanation:** The conclusions summarize the study’s key findings well and highlight important research gaps. The identification of future research directions is particularly useful, as it provides a roadmap for scholars interested in AI and finance. However, while the paper discusses research gaps and trends, it does not sufficiently address practical implications for financial institutions, policymakers, or industry practitioners. Given the relevance of AI in real-world financial applications, a discussion on how financial institutions can use these insights would enhance the paper’s impact. Additionally, the ethical implications of AI in finance—such as bias in AI-driven decision-making and the risks of algorithmic trading—are briefly mentioned but not explored in depth.

**Recommendation:**

 Expand the discussion on practical implications for financial decision-makers and policymakers. Provide a clearer link between the identified research gaps and potential industry applications.

Is the work clearly and accurately presented and does it cite the current literature?

Yes

If applicable, is the statistical analysis and its interpretation appropriate?

Yes

Are all the source data underlying the results available to ensure full reproducibility?

Yes

Is the study design appropriate and is the work technically sound?

Yes

Are the conclusions drawn adequately supported by the results?

Partly

Are sufficient details of methods and analysis provided to allow replication by others?

Partly

Reviewer Expertise:

Tourism Psychology, Tourism Marketing, Sustainable tourism Practice, Tourism & AI, Tourism and Machine Learning.

I confirm that I have read this submission and believe that I have an appropriate level of expertise to confirm that it is of an acceptable scientific standard.

# Reviewer report: Reviewer response for version 1

**DOI:** 10.5256/f1000research.176927.r362857

**Published:** 2025-02-03

**Authors:** Saad Salih Hussein

**Affiliations:** 1 Accounting, Tikrit University, Tikrit, Saladin Governorate, Iraq; 2 Tikrit University, Tikrit, Iraq

This article discusses a new aspect of the relationships between Artificial intelligence and finance, it gives, therefore, a rule of thump to the literature on accounting and finance, the researchers have used a sufficient amount of background articles to support their argument to prove the relationship between the main variables of this study, it is thus, this article has written carefully in the right path with all supported evidence to complete the paper argument.

**Summary of the Article**

 The article provides a comprehensive bibliometric analysis of the intersection of artificial intelligence and finance, shedding light on its evolution, central themes, and potential research avenues which is welcoming. The study uses 607 Web of Science indexed papers and follows the Theory-Context-Characteristics-Methodology (TCCM) framework which is commendable. The research highlights key themes such as risk management, market efficiency, innovation, and emerging areas like ethical AI and AI-driven financial decision-making. The findings emphasize critical gaps in interdisciplinary methodologies, ethical considerations, and the need for more transparent AI systems in financial applications.

**Strengths of the Article**

 Comprehensive Scope – The study successfully maps the evolution of AI in finance, providing a well-structured thematic analysis.

 Robust Methodology – The use of bibliometric techniques and the TCCM framework adds rigor to the research, ensuring a systematic approach.

 Relevance to Academia and Industry – The findings are valuable for researchers, practitioners, and policymakers, offering insights into the transformative role of AI in finance.

 Identification of Key Gaps – The study highlights crucial research gaps, particularly in ethical AI and methodological advancements, making it a strong foundation for future research.

**Major Points for Improvement**

 1. Expanding Practical Implications through empirical research– The findings highlight key trends in AI adoption within finance; however, further elaboration on the direct industry applications of these insights would enhance the study’s practical relevance. Providing specific examples of AI’s role in areas such as credit risk assessment, fraud detection, portfolio management, and regulatory compliance could help bridge the gap between research and practice.

**Minor Points for Improvement**

 1. Incorporating Recent Literature – The study is well-grounded in existing research; however, integrating more recent publications (2023–2025) would further enhance its timeliness. Including the latest studies on AI applications in finance, particularly in ethical AI and regulatory advancements, would ensure the findings remain current and relevant.

 Nevertheless, the article can be accepted for indexing in bibliographic databases.

Is the work clearly and accurately presented and does it cite the current literature?

Yes

If applicable, is the statistical analysis and its interpretation appropriate?

Yes

Are all the source data underlying the results available to ensure full reproducibility?

Yes

Is the study design appropriate and is the work technically sound?

Yes

Are the conclusions drawn adequately supported by the results?

Yes

Are sufficient details of methods and analysis provided to allow replication by others?

Yes

Reviewer Expertise:

the systematic way of writing to present the researchers' ideas was credit for the paper

I confirm that I have read this submission and believe that I have an appropriate level of expertise to confirm that it is of an acceptable scientific standard.
